# Microwave-Assisted Extraction of Herbacetin Diglucoside from Flax (*Linum usitatissimum* L.) Seed Cakes and Its Quantification using an RP-HPLC-UV System

**DOI:** 10.3390/molecules19033025

**Published:** 2014-03-10

**Authors:** Ophélie Fliniaux, Cyrielle Corbin, Aina Ramsay, Sullivan Renouard, Vickram Beejmohun, Joël Doussot, Annie Falguières, Clotilde Ferroud, Frédéric Lamblin, Eric Lainé, Albrecht Roscher, Eric Grand, François Mesnard, Christophe Hano

**Affiliations:** 1 Laboratoire de Phytotechnologie EA 3900 BIOPI, Faculté de Pharmacie, Université de Picardie Jules Verne, 1 rue des Louvels, 80037 Amiens, France; E-Mails: ophelie.fliniaux@u-picardie.fr (O.F.); aina.ramsay@u-picardie.fr (A.R.); sullivan.renouard@univ-orleans.fr (S.R.); vickram.beejmohun@u-picardie.fr (V.B.); 2 Laboratoire de Biologie des Ligneux et des Grandes Cultures UPRES EA 1207, Equipe Lignanes des Linacées, Université d’Orléans — Antenne Scientifique Universitaire de Chartres, 21 rue de Loigny la Bataille, 28000 Chartres, France; E-Mails: cyrielle.corbin@univ-orleans.fr (C.C.); joel.doussot@cnam.fr (J.D.); frederic.lamblin@univ-orleans.fr (F.L.); eric.laine@univ-orleans.fr (E.L.); christophe.hano@univ-orleans.fr (C.H.); 3 Génie Enzymatique et Cellulaire, FRE 3580 CNRS, Université de Picardie Jules Verne, 33 rue Saint-Leu, 80039 Amiens, France; E-Mail: albrecht.roscher@u-picardie.fr; 4 Laboratoire des Glucides FRE 3517 CNRS, Faculté des Sciences, Université de Picardie Jules Verne, 33 rue Saint-Leu, 80039 Amiens, France; E-Mail: eric.grand@u-picardie.fr; 5 Ecole SITI (Département CASER), Conservatoire National des Arts et Métiers, 292 rue Saint Martin, 75141 Paris Cedex 03, France; 6 Service de Transformations Chimiques et Pharmaceutiques, ERL CNRS 3193, Conservatoire National des Arts et Métiers, 2 rue Conté, 75003 Paris, France; E-Mails: annie.falguieres@cnam.fr (A.F.); clotilde.ferroud@cnam.fr (C.F.)

**Keywords:** flavonol, flaxseed, herbacetin diglucoside, *Linum usitatissimum*, microwave-assisted extraction

## Abstract

Flax (*Linum usitatissimum* L.) seeds are widely used for oil extraction and the cold-pressed flaxseed (or linseed) cakes obtained during this process constitute a valuable by-product. The flavonol herbacetin diglucoside (HDG) has been previously reported as a constituent of the flaxseed lignan macromolecule linked through ester bonds to the linker molecule hydroxymethylglutaric acid. In this context, the development and validation of a new approach using microwave-assisted extraction (MAE) of HDG from flaxseed cakes followed by quantification with a reverse-phase HPLC system with UV detection was purposed. The experimental parameters affecting the HDG extraction yield, such as microwave power, extraction time and sodium hydroxide concentration, from the lignan macromolecule were optimized. A maximum HDG concentration of 5.76 mg/g DW in flaxseed cakes was measured following an irradiation time of 6 min, for a microwave power of 150 W using a direct extraction in 0.1 M NaOH in 70% (*v/v*) aqueous methanol. The optimized method was proven to be rapid and reliable in terms of precision, repeatability, stability and accuracy for the extraction of HDG. Comparison with a conventional extraction method demonstrated that MAE is more effective and less time-consuming.

## 1. Introduction

Flax (*Linum usitatissimum* L., Linaceae) is a common oilseed crop regarded as a functional food that constitutes a key source of phytochemicals [1]. During the last decade, there has been an increasing interest in the human consumption of flaxseed in the diet in order to improve nutritional and health status [1]. Flaxseed is rich in oil with a very high α-linolenic acid (omega 3 fatty acid) content; it also contains a high level of dietary fiber and good quality protein fractions [1,2].

**Figure 1:**
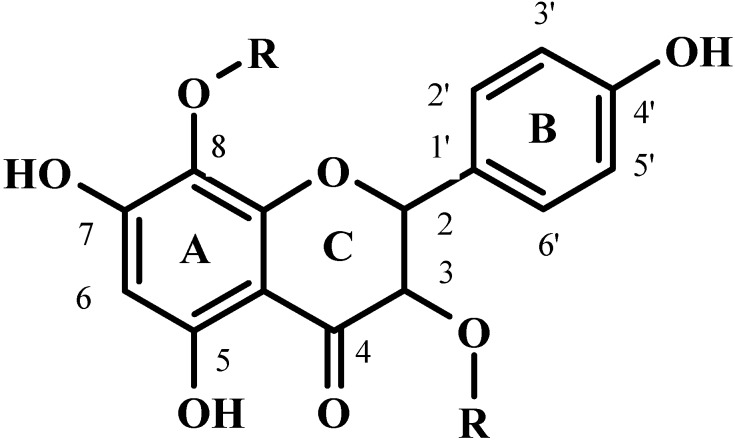
Structures of herbacetin (R = H) and herbacetin diglucoside (HDG; R = glucose).

Flaxseed hulls represent a rich source of valuable metabolites such as lignans [3,4,5,6], hydroxycinnamic acids (HCA) [4,5] and flavonols [4]. The main flaxseed lignan, secoisolariciresinol diglucoside (SDG), has been shown to reduce the incidence of a wide variety of cancers, lower the risk of cardiovascular diseases, limit hypercholesterolemic atherosclerosis and delay the development of diabetes [7,8,9,10]. However, the possibility that other phytochemicals accumulated in flaxseed hulls also contribute to the health benefits ascribed to flaxseed cannot be excluded. In particular, attention may be paid to the flavonol herbacetin diglucoside (HDG; Figure 1) which has been recently demonstrated to be a constituent of the lignan macromolecule of flaxseed hulls, linked through ester bonds to hydroxymethylglutaric acid (HMG), together with SDG and HCA glucosides (HCAG) [4].

Flavonols present a large spectrum of biological activities, being the most active compounds within the flavonoid group. In fact, the beneficial effects on cardiovascular health of diets rich in fruits and vegetables have been attributed to flavonoids in general and often to flavonols in particular. The major dietary flavonols studied are quercetin, kaempferol, myricetin, and isorhamnetin. Epidemiological studies have shown an inverse correlation between flavonol intake and coronary heart disease and stroke [11]. Human intervention trials with isolated flavonols have demonstrated an anti-hypertensive effect. Besides their high antioxidant capacity, flavonols are also known to interact with estrogenic receptors [12]. Various studies have shown that flavonols have properties that alleviate several diseases, including inflammation and cancer [13]. The potential involvement of herbacetin in the stimulation of renal tubular epithelial cells, improvement of renal function, treatment of renal failure and promotion of bone formation has been mentioned in a patent claim [14]. Moreover *in vitro* anti-influenza viral activity through neuraminidase inhibition has been evidenced [15]. Therefore, HDG could contribute (perhaps synergistically with other flaxseed hull constituents) to the health benefits ascribed to flaxseed. To date, there is little information available concerning herbacetin’s biological activities.

Microwave-assisted extraction (MAE) has the advantage of being conducted rapidly and generally offers a significant improvement in terms of both extraction time and solvent consumption [5]. The occurrence of HDG in flaxseed hulls has already been reported [4,16], however the extraction protocols merit optimization. Interestingly, flaxseed cakes constitute a hull-enriched by-product of the oil industry and could therefore be an attractive source for the production of HDG. Therefore, the aim of the present study was to develop and validate an efficient MAE protocol for the quantification of HDG in flaxseed cakes and to compare it with a more conventional solid/liquid extraction procedure, which is a heat reflux extraction described in Section 3.4.

## 2. Results and Discussion

### 2.1. Optimization of Microwave-Assisted Extraction

Struijs *et al.* [4] demonstrated that HDG is a constituent of the flaxseed lignan macromolecule. The release of the components of this macromolecular complex is generally achieved by sequential or simultaneous alcoholic solid-liquid extraction and alkaline treatment (for a review, see [6]). For this purpose, sodium hydroxide concentrations ranging from 0.1 M [3] to 1 M [17] are commonly employed. The objective of the present study was to develop a rapid extraction process for the quantification of HDG in flaxseed products using microwaving.

The chromatographic separation used in this study was achieved using a reverse-phase C18 column, a nonlinear gradient of acetic acid acidified-water and methanol and a column temperature set at 35 °C. Identification of HDG was based on a comparison of retention time, UV and MS spectra (*m*/*z* = 625.08 [M−H]^−^) with those of the commercial standard of HDG (*m*/*z* = 625.05 [M−H]^−^) as well as by standard addition. According to the maximum absorption wavelength of HDG, the detection wavelength for quantization was set at 280 nm. The quantization method was developed using *o*-coumaric acid as internal standard.

Factorial experiment design and response surface plot methodology were used to identify the relationship between the extraction parameters, as well as the response functions and the process variables, in order to determine the optimized extraction conditions. Three independent variables were studied: extraction time (X_1 _values were 1, 6 and 15 min), microwave power (X_2_ values were 50, 100 and 150 W) and NaOH concentration (X_3_ values were 0.1 and 1 M) (Table 1). These values have been chosen in relation with a previous study on the use of MAE for other phenolic compounds [5].

**Table 1:** Coded levels and experimental values of the 3 independent variables.

Independent variable	Code unit	Coded variable levels		
		−1	0	+1
Time (min)	X_1_	1	6	15
Microwave power (W)	X_2_	50	100	150
NaOH concentration (M)	X_3_	0.1	-	1

The values of the independent process variables considered are given in Table 2. 

**Table 2:** Experimental design using Box-Behnken. Values are the mean ± RSD of 3 independent replicates.

Batch	X_1_	X_2_	X_3_	X_1_^2^	X_2_^2^	X_3_^2^	X_1_ X_2_	X_2_ X_3_	X_1_X_3_	X_1_ X_2_ X_3_	Quantified HDG (mg/g DW)
1	−1	−1	−1	1	1	1	1	1	1	−1	2.01 ± 0.49
2	−1	−1	1	1	1	1	1	−1	−1	1	1.75 ± 0.24
3	−1	0	−1	1	0	1	0	0	1	0	3.42 ± 0.46
4	−1	0	1	1	0	1	0	0	−1	0	4.27 ± 0.09
5	−1	1	−1	1	1	1	−1	−1	1	1	4.80 ± 0.21
6	−1	1	1	1	1	1	−1	1	−1	−1	5.00 ± 0.24
7	0	−1	−1	0	1	1	0	1	0	0	2.90 ± 0.01
8	0	−1	1	0	1	1	0	−1	0	0	3.85 ± 0.11
9	0	0	−1	0	0	1	0	0	0	0	4.35 ± 0.23
10	0	0	1	0	0	1	0	0	0	0	4.56 ± 0.13
11	0	1	−1	0	1	1	0	−1	0	0	5.76 ± 0.47
12	0	1	1	0	1	1	0	1	0	0	4.15 ± 0.13
13	1	−1	−1	1	1	1	−1	1	−1	1	2.81 ± 0.20
14	1	−1	1	1	1	1	−1	−1	1	−1	2.40 ± 0.04
15	1	0	−1	1	0	1	0	0	−1	0	3.67 ± 0.23
16	1	0	1	1	0	1	0	0	1	0	4.23 ± 0.23
17	1	1	−1	1	1	1	1	−1	−1	−1	5.20 ± 0.10
18	1	1	1	1	1	1	1	1	1	1	4.37 ± 0.34

**Figure 2:**
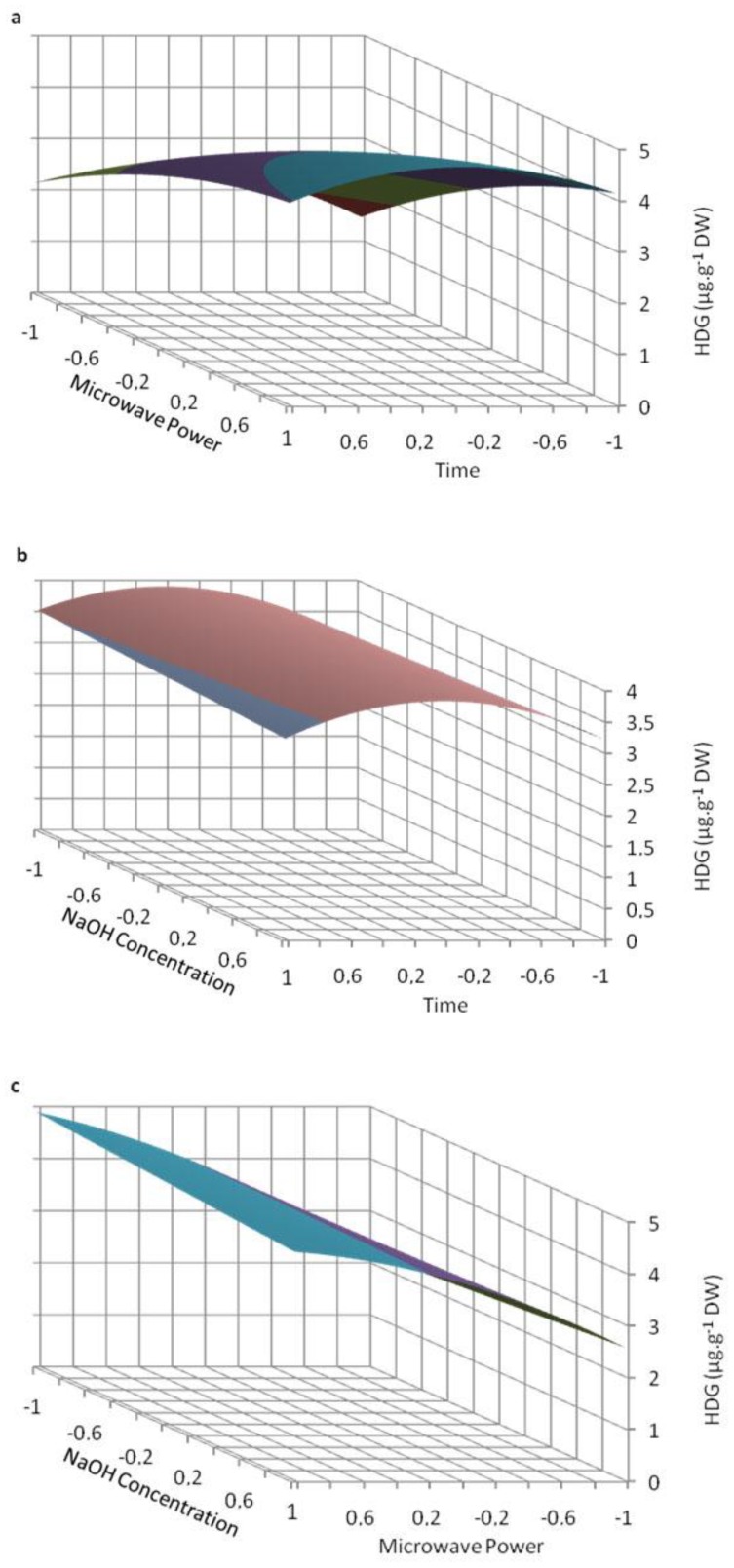
Predicted surface response plots of the HDG extraction yield as a function of (**a**) microwave power and time, (**b**) NaOH concentration and time, and (**c**) NaOH concentration and microwave power.

The following second-order polynomial equation was fitted to the experimental values by multiple regressions: Y = 3.86 + 0.12X_1_ + 1.13X_2_ − 0.02X_3_ − 0.6X_1_^2^ − 0.33X_2_^2^ − 0.21X_1_X_2_ − 0.21X_2_X_3_ − 0.12X_1_X_3_ − 0.11X_1_X_2_X_3_. The quality of fit was checked using the coefficient of determination (R^2^). Its value (0.874) indicates a relatively satisfactory agreement between the measured and the predicted responses. All these trends were recorded in three-dimensional response surface plots, using the determined polynomial equation (Figure 2). 

The best HDG yield was both predicted and measured for 6 min of irradiation using 150 W of microwave power and 0.1 M NaOH concentration (Table 2). For the shortest incubation time (1 min), sodium hydroxide concentration appeared to be a determinant parameter for MAE of HDG from the flaxseed lignan macromolecule. In fact, the results clearly demonstrated that, whatever the microwave power used for such a short time, a sodium hydroxide concentration of 1.0 M always allowed a better HDG release than 0.1 M (Figure 2b; Table 2). In a previous work, sodium hydroxide concentration was evidenced as a determinant parameter for the extraction yield of hydroxycinnamic acid glucosides but not for SDG extracted from the flaxseed lignan macromolecule [5]. The influence of sodium hydroxide concentration for a short MAE time might be explained by a stronger binding between HDG and HMG than between SDG and HMG in the lignan macromolecule or by a different solubility of these compounds in the solvent system.

The influence of microwave power was also determinant since for 1 min of irradiation the HDG yield measured at 50 W was only about 50% of that at 100 W and about 40% of that at 150 W (Figure 2a,b; Table 2), and this was independent of sodium hydroxide concentration. The HDG yield increased with incubation time from 1 to 6 min, reaching a maximum value after 6 min of microwaving in every condition, except for 150 W and 1.0 M NaOH when the maximum occurred after 1 min of irradiation (Figure 2; Table 2). 

The coefficients of the second-order polynomial equation obtained for extraction time and microwave power were positive, indicating that increasing these parameters favored HDG extraction overall, as previously noted for other natural products, such as fucocoumarins or phenolics [18,19]. However, considering the negative coefficient obtained for X_1_^2^ (X_1_, time), it clearly appears that HDG extraction according to this parameter passes by a maximum (Figure 2a,b; Table 2) for this parameter. For each condition, prolonging extraction time, particularly with 1.0 M sodium hydroxide and microwave power of 150 W, resulted in a decreased HDG yield. When the microwave power was 150 W, using 0.1 M NaOH, the highest HDG extraction yield was obtained for the intermediate time. Prolonging extraction time in these conditions caused a decrease of 10% of the maximum obtained (5.20 *vs.* 5.76). When the microwave power was 150 W, using 1.0 M NaOH, the highest HDG extraction yield was obtained for the shortest time. Prolonging extraction time in these conditions caused a decrease of 13% of the maximum obtained (4.37 *vs*. 5.00) (Figure 2a,b; Table 2). This decrease observed when prolonging time could be due to partial degradation of HDG. Microwave irradiation has been already described to induce oxidation of natural products such as olive oil [20], even if it occurred after longer experiment time (hours *vs.* minutes).

### 2.2. Method Validation

The MAE and the HPLC method were validated to ensure the precision, repeatability, stability and accuracy of the method developed to extract and quantify HDG. The linear correlations between peak area and standard concentrations were found to be high in the range of 5 to 1,000 µg/mL for HDG. The resulting linear equation and R^2^-value for a 10-point calibration graph were > 0.999, and the slope of five replicates of the calibration graph covering the analytical range for the HDG standard varied no more than 1% in terms of RSD over a period of four weeks. The LOD (S/N = 3) and LOQ (S/N = 10) were 0.28 and 0.92 µg/mL, respectively. The RSD of retention times during the validation procedure were satisfactory (0.46%; Table 3).

**Table 3:** Analytical performance of the proposed method. Values are the mean (± SE) of 5 independent replicates.

Retention time (min)	Calibration	R^2^	Linear range (µg/mL)	LOD (µg/mL)	LOQ(µg/mL)
26.26 ± 0.12	y = 1.429x − 1.312	0.9994	5–1000	0.28	0.92

Table 4 shows the results for precision, repeatability and stability of the optimized extraction protocol. In order to evaluate the instrumental precision, five injections of the same sample were performed. The chromatographic method was proved to be precise with an RSD of 0.42% (Table 4). Repeatability was evaluated by applying the whole extraction procedure three times to the same batch of material and the obtained RSD value was low (0.98%; Table 4). The stability was determined by six injections of the same sample during 72 h (0, 6, 12, 24, 48 and 72 h after it was prepared). A good stability of the extracted sample was observed with a low RSD value of 0.26% (Table 4). 

**Table 4:** Analytical results of precision, repeatability and stability tests. HDG contents (in flaxseed cake extract obtained using optimal extraction parameters) are the mean ± RSD of n independent replicates.

Precision (*n* = 5)		Repeatability (*n* = 3)		Stability (*n* = 6)	
Content (µg/mL)	RSD (%)	Content (µg/mL)	RSD (%)	Content (µg/mL)	RSD (%)
21.44 ± 0.09	0.42	19.60 ± 0.24	0.98	19.48 ± 0.05	0.26

The accuracy of the separation method was evaluated by the standard addition procedure with three addition levels of 5, 10 and 15 µg/mL of HDG (Table 5). The results demonstrated a good recovery of the compound ranging from 99.60% to 99.88% with low RSD values ranging from 0.37% to 1.38% (Table 5).

**Table 5:** Accuracy test determined by the standard addition method at three added concentrations HDG contents (in flaxseed cake extract obtained using optimal extraction parameters) are the mean ± RSD of 3 independent replicates.

Spike concentration (µg/mL)	In sample (before addition) (µg/mL)	Expected (calculated) (µg/mL)	Actual (measured)(µg/mL)	Recovery (%)	RSD (%)
5	19.60 ± 0.24	24.60	24.57 ± 0.34	99.88	1.38
10	19.60 ± 0.24	29.60	29.48 ± 0.37	99.60	1.25
15	19.60 ± 0.24	34.60	34.50 ± 0.13	99.71	0.37

### 2.3. Comparison with Conventional Liquid-Solid Extraction

An analysis of HDG extraction efficiency from flaxseed cakes comparing conventional heat reflux extraction with MAE under the optimal conditions (extraction time of 6 min at 150 W with 0.1 M sodium hydroxide giving a maximum HDG yield of 5.76 mg/g DW) is presented in Table 6. The results clearly indicate that MAE gives a higher HDG yield and in a shorter extraction time than does the conventional method. In fact, in less than 30 min, no HDG could be detected using the conventional heat reflux extraction method, while after 60 min of extraction time, the HDG yield reached only 45% of that obtained for MAE (Table 6). It is of particular interest within the context of green chemistry, especially in term of reducing energy consumption by using innovative technologies and of valorization of by-products since flaxseed cakes result from the production of flax oil [21]. MAE enables heating of the solvent mixture by directly interacting with the free water molecules present in the system, which results in the rupture of the plant tissue and release of the constituents into the solvent. MAE commonly increases phenolic yields and lowers extraction costs due to the reduction of treatment time and solvent consumption [22]. 

**Table 6:** Comparison between conventional and microwave-assisted extraction of herbacetin diglucoside using 0.1 M NaOH. nd: not detected. Values are the mean ± RSD of three independent replicates and different letters (a, b, c) indicate significant differences between conditions (*p* < 0.05).

	Conventional heat reflux extraction					MAE 150 W
Time (min)	1	6	15	30	60	6
HDG (mg/g DW)	nd	nd	nd	1.90 ± 0.28 ^a^	2.60 ± 0.48 ^b^	5.76 ± 0.47 ^c^

In the literature, Struijs *et al.* [4], using a hydroalcoholic extraction with 63% (*v/v*) aq. EtOH followed by a 24-h alkaline hydrolysis treatment (NaOH, 75 mM), reported an HDG yield of 2 mg/g DW measured in flaxseed cake (thus comparable to our conventional extraction yield) whereas Qiu *et al.* [16], using whole flaxseed as the raw starting material and acidic extraction conditions, reported a much lower HDG content (0.01% w/w DW) which may have been due to acid hydrolysis of HDG: deglucosylation followed by the formation of a reactive herbacetin carbocation as suggested by Struijs *et al.* [4]. 

Presence of herbacetin or differently glycosylated derivatives has been reported in a few other plants such as *Ephedra* [23], Ramose Scouring Rush herb [24], *Sedum hybridum* [25], or *Rhodiola* species [26,27,28]. Quantification was only performed for *Rhodiola rosea*, herbacetin derivative are present in roots at a content of 3 mg/g DW, which is lower yield than that obtained in this study.

## 3. Experimental

### 3.1. Chemicals and Standards

All solvents and reagents for extraction and HPLC analysis were of analytical grade (ACS) or the highest available purity and were purchased from Fisher Scientific (Illkirch, France). Deionized water was purified by a Milli-Q water-purification system from Millipore (Molsheim, France). All solutions prepared for HPLC were filtered through 0.45 µm nylon syringe membranes prior to use. The HDG standard was purchased from Chromadex (Molsheim, France). and *o*-coumaric acid (internal standard) was purchased from Sigma (Saint-Quentin Fallavier, France).

### 3.2. Plant Material

Cold-pressed flaxseed cake (*Linum usitatissimum* L., cultivar Barbara), provided by the “Centre de Valorisation des Glucides et des Produits Naturels” (Amiens, France) was used for the development and validation of the extraction method.

### 3.3. Microwave-Assisted Extraction

MAE was performed using a Discover microwave system (CEM, Orsay, France), which operates at a maximum power of 300 W. The sample (500 mg) was placed in a 100-mL quartz tube topped by a vapor condenser and was suspended in 20 mL 70% (*v/v*) aq MeOH supplemented with 0.1 M or 1.0 M sodium hydroxide. The different powers used were 50, 100 and 150 W and the extraction times were 1, 6 and 15 min. The extract was then neutralized with acetic acid, centrifuged for 15 min at 3,000 rpm and the supernatant was filtered (0.45 µm) before HPLC analysis.

### 3.4. Conventional Solid/Liquid Extraction

The conventional heat reflux extraction was adapted from [3]. The sample (500 mg) was suspended in 20 mL 70% (*v/v*) aq MeOH supplemented with 0.1 M sodium hydroxide in a 100-mL tube topped by a vapor condenser and placed in a hot water bath at a temperature of 50 °C for different extraction times from 1 to 60 min. The extract was then neutralized with acetic acid, centrifuged for 15 min at 3,000 rpm and the supernatant was filtered (0.45 µm) before HPLC analysis.

### 3.5. RP-HPLC Analysis

The determination of HDG concentration (content, or quantification) was carried out on a Varian liquid chromatographic system (Agilent, Les Ulis, France) including a Varian Prostar 230 pump, a Metachem Degasit degasser, a Varian Prostar 410 autosampler and a Varian Prostar 335 Photodiode Array (PDA) detector and controlled by Galaxie version 1.9.3.2 software. The separation was performed at 35 °C on a Purospher (Merck, Fontenay Sous Bois, France).) RP-18 column (250 × 4.0 mm i.d.; 5 µm).

The mobile phase consisted of 0.2% acetic acid in water (solvent A) and methanol (solvent B). The composition of the mobile phase varied during the run according to a nonlinear gradient at a flow rate of 0.8 mL/min as follows: from 0–40 min of A-B: 90:10 (*v/v*) to 30:70 (*v/v*), from 41–50 min of A-B: 30:70 (*v/v*) to 0:100 (*v/v*), and A-B: 0:100 (*v/v*) from 51–60 min. 

Detection was performed at 280 nm. HDG was identified by comparison with an authentic standard (Chromadex) and quantified against a 10-point calibration curve ranging from 5 µg/mL to 1 mg/mL (y = 1.429x − 1.312) with a correlation coefficient of 0.9994 and using *o*-coumaric acid as an internal standard (0.05 mg/mL).

### 3.6. LC-MS Analysis

LC-MS analyses were performed on a Waters 2695 Alliance coupled with a single quadrupole mass spectrometer ZQ (Waters-Micromass, Manchester, UK) equipped with an electrospray ion source (ESI-MS). The chromatography was carried out using the conditions described above. Reference compounds and raw cell extracts were loaded on the KROMASIL column using a sample injection volume of 20 µL (methanol solutions at 0.1 g/L for the reference compound and at 1 g/L for the crude extracts). The effluent was flow-split via a PEEK tee with 1/5 of the flow directed towards the ESI source of the ZQ instrument and the residual 4/5 directed towards a PDA detector (Waters 2996). 

LC-ESI-MS data were recorded in the positive and negative ion modes. The source and desolvation temperatures were kept at 120 and 250 °C, respectively. Nitrogen was used as a drying and nebulizing gas at flow rates of 450 and 100 L/h, respectively. The capillary voltage was ±3.5 kV and a cone voltage ranging from ±20 to ±60 V was used (±ESI). Scanning was performed in the range of 50–1950 Da at a scan rate of 1 s per scan. Data were collected in the centroid mode. Data acquisition and processing were performed with MassLynx V4.0 software.

### 3.7. Experimental Design

Factorial experiment design and response surface plots were selected to find the optimal HDG extraction conditions using XLSTAT2012 software (Addinsoft, Paris, France). Variables were coded at two or three levels (−1 and +1 or −1, 0 and +1; Table 1). These three independent variables (see Table 1) were extraction time (X1 values were 1, 6 and 15 min), microwave power (X2 values were 50, 100 and 150 W) and NaOH concentration (X3 values were 0.1 and 1 M). Eighteen batches of different combinations were prepared by taking values of selective variables at different levels as shown in Table 2. The experiments were carried out in triplicate. The three independent variables were coded according to the following equation xi = (Xi − X0)/ΔXi, with i = 1, 2, 3 and where xi and Xi are the dimensionless and the actual value of the independent variable i, respectively, X0 is the actual value of the independent variable i at the central point, and ΔXi is the step change in Xi corresponding to a unit change in the dimensionless value. The response at each design point was recorded. Data from the central composite experimental design were subjected to regression analysis using least-square regression methodology to obtain the parameters of the mathematical model. Student’s t-test was used to check the statistical significance of the regression coefficients derived from the model. Analysis of variance (ANOVA) was applied to evaluate the statistical significance of the model. Surface plots showing the response as a function of the simultaneous variation of the independent variables were obtained using the fitted model.

### 3.8. Statistical Treatment of Data

All data presented in this study are the mean and the standard deviation of at least three independent replicates. Comparative statistical analyses of groups were performed using one-way analysis of variance (ANOVA). All statistical tests were considered significant at *p* < 0.05.

## 4. Conclusions

Herbacetin diglucoside has already been demonstrated to be a promising compound. Nevertheless, its extraction was far from efficient, thus restricting the availability of enough HDG for further investigation of its biological activities. The present study describes an efficient and validated MAE method for HDG extraction and quantification from cold-pressed flaxseed cakes, confirming that this oil industry by-product can be used as the raw starting material for the extraction of valuable secondary metabolites. A maximum HDG concentration of 5.76 mg/g DW in flaxseed cakes was measured following an irradiation time of 6 min, with a microwave power of 150 W using a direct extraction in 0.1 N NaOH in 70% (*v/v*) aqueous methanol. The proposed method is more efficient and less time-consuming than the conventional method. It is clearly confirmed as suitable for HDG extraction in terms of precision, repeatability, stability and accuracy. It will allow fast and easy quantification of HDG in a number of flax samples (different cultivars or derived food). Moreover, this improved extraction will facilitate *in vivo* and *in vitro* experiments aimed at elucidating the biological activity of this promising compound.
